# We do as we construe: extended behavior construed as one task is executed as one cognitive entity

**DOI:** 10.1007/s00426-018-1051-2

**Published:** 2018-07-18

**Authors:** Ausaf A. Farooqui, Tom Manly

**Affiliations:** MRC-Cognition and Brain Sciences Unit, 15 Chaucer Road, Cambridge, CB2 7EF UK

## Abstract

We select and execute extended task episodes (‘make tea’) as one entity and not individually execute their very many components (find kettle, boil water, etc.). Such hierarchical execution is thought to occur in familiar task situations with pre-existing task episode-related scripts that once selected, control the identity and sequence of component steps. Here, in contrast, we show hierarchical execution of extended behavior in situations, where the identity and sequence of component steps were unknown and a predetermined script could not have existed. Participants performed a rule-switching task in which the rule to be applied on each trial could not be predicted. Crucially, they were biased into construing a recurring instance of three or five trials as a *single task episode*. Behavioral signs of hierarchical execution, identical to those seen during memorized task-sequence executions, were present. These included longer reaction time on the first trial of each episode that was proportionate to the length of that episode, and absence of rule switch costs only between those consecutive trials that crossed episode boundaries. Hierarchical execution thus occurs every time the to-be-executed behavior is construed as one task episode, and is not limited to predictable sequences. We suggest that hierarchical execution occurs because task episodes are controlled and executed through goal-related entities assembled at the beginning of execution that subsume the execution and instantiate purposive control across time until the goal is complete.

## Introduction

Goal-directed behavior frequently consists of temporally extended actions and task entities (task episodes, sub-episodes) that consist of a sequence of steps (e.g., sub-tasks, smaller acts and events etc.), but are nonetheless executed as one entity. The various components of checking email at different levels of detail (‘open browser’, ‘move cursor’, ‘click’) or that of preparing breakfast are executed as one entity (e.g., Botvinick, Niv, & Barto, 2009; Dezfouli, Lingawi, & Balleine, 2014; Logan, 1988). Nonetheless, how the sequence of acts corresponding to such task episodes constitute as one cognitive entity is unclear. More so, because what constitutes a task is often determined by the doer’s construal and not by the physical environment (Vallacher & Wegner, 1989). The same breakfast, for example, can be prepared as one (‘prepare breakfast’), two (‘prepare tea’ and ‘prepare toast’), or four task episodes (‘boil water’, ‘brew tea’, ‘toast bread’, and ‘spread butter’).

The experiments presented here examine the cognitive underpinnings of task episodes. We use the term *task episodes*, because these typically are temporally extended periods of focused purposive behavior during which a sequence of constituent steps is executed. Here, while participants executed continuous trials of an experimental session, task-irrelevant cues were used to bias them towards viewing each recurring period of three or five trials as *one* task episode. This method allowed us to study factors related to task episodes without confounding them with factors pertaining to task rules, long-term memory recall, working memory, attention, action selection etc. While, for simplicity, we use the phrase ‘task episode’, we do not insist that in the task hierarchy, these necessarily are tasks as opposed to sub-tasks of a larger task. Our concern is that irrespective of the hierarchical level, the sequence of trials be construed and executed as one entity.

### Hierarchical nature of actions

Almost all task and action constructs (e.g., ‘prepare breakfast’, ‘write email’, etc.) through which we execute our behavior correspond to extended task episodes (see Vallacher & Wegner, 1987). The selection, instantiation, and execution of such task episodes as one entity in spite of their consisting of a sequence of smaller acts and processes make our actions hierarchical. An extended episode of thought and behavior can be selected and executed as one unit only through a cognitive entity that can be selected and instantiated as one, but corresponds to or controls the extended episode of thought and behavior. Existing account of hierarchical cognition recognize the presence of such cognitive entities only in predictable situations, where the knowledge (both procedural and declarative) related to the identity and sequence of component steps is accessible as a single mnemonic representational entity described variously as schemas, scripts, frames, plans (Miller, Galanter, & Pribram, 1960; Minsky, 1974; Norman & Shallice, 1986; Rumelhart & Norman, 1982; Schank & Abelson, 1977). Being related to the knowledge of what is to be done when in the task episode, these entities control the identity and sequence of the component steps much like a recipe controls the preparation of a dish. For example, the schema about preparing coffee may consist of ‘add coffee from packet’, ‘add sugar from bowl’, ‘add milk from carton’, each of which may have their own sub-schemas. The main schema remains active across time and sequentially selects the lower level schemas.

Execution of such predictable behavioral sequences generates signs of hierarchical execution. When participants recall or execute a memorized sequence of motor or task items (Anderson et al., 1998; Kahana & Jacobs, 2000; Lien & Ruthruff, 2004; Rosenbaum, Kenny, & Derr, 1983; Schneider & Logan, 2006): (1) item 1 reaction time (RT) tends to be the longest; (2) this item 1 RT is frequently longer for longer/more complex sequences, e.g., it takes longer to start speaking longer words (Klapp, Anderson, & Berrian, 1973), and start executing task lists with more item-level switches (Desrochers, Chatham, & Badre, 2015; Schneider & Logan, 2006); (3) when a sequence of more than one task item is being executed, then the behavioral cost of switching (or benefits of repeating) tends to be absent when component items switch (or repeat) across sequence boundaries (Lien & Ruthruff, 2004; Schneider & Logan, 2006, 2015). For example, in a switching task that sometimes requires color judgments (C) and sometimes judgments of shape (S), trials that require a different judgment from the previous trials (CS or SC) would be expected to have significantly higher error rates and RTs than trials that repeat the previous rule (CC or SS). Executing a memorized sequence like CCSS will result in a switch across the sequence boundary (CCSS–CCSS–CCSS), while executing a sequence like CSSC will not (CSSC–CSSC–CSSC). Despite this, accuracy and RT on item 1 of CCSS tend not to be higher than CSSC (Schneider & Logan, 2006).

These three signs of hierarchy have been interpreted as resulting from the dynamics of recall and instantiation of a hierarchical task-sequence representation (e.g., the memorized sequence CCSS) in working memory that controls the identity and sequence of component steps (e.g., Mayr, 2009; Perlman, Pothos, Edwards, & Tzelgov, 2010; Schneider & Logan, 2006, 2015). Beginning to execute the sequence requires instantiating the task-sequence representation in working memory, hence the longer item 1 RT, and longer sequences take longer to instantiate causing longer item 1 RTs for longer sequences. Control processes instantiating a hierarchical sequence structure in working memory have a hierarchical relation with the control processes related to the lower level task item (e.g., switch control), and hence, their instantiation at the beginning also suffices for switch control at item 1 (Schneider & Logan, 2006, 2015).

To our knowledge, these explanations have not, however, been tested in the context of task episodes that are unpredictable, where there can be no memory of the order in which different processes must be performed. This is interesting, because in everyday life, many activities in which we engage have no fixed script; we may shop without a list in mind or engage in conversations the content of which cannot be anticipated, but, nevertheless, reliably identify the corresponding collection of behavior as one task (Vallacher & Wegner, 1987). Similarly, even apparently routine tasks often require much flexibility in the precise actions and order of actions that will be required (the cup is missing, and the tea caddy is empty). Of course, whether the single ‘task’ breaks down into new sub-tasks when such impasses are reached or whether our characterization of a period of behavior as a single task is merely a linguistic label is unclear. In this respect, the reliable presence of the signs highlighted above during unpredictable sequences construed as a single task would indicate a higher level organizing cognitive entity that operates despite an inability to precisely anticipate when given processes should be deployed. One possibility is that this entity may instantiate various goal-related control processes needed for searching the unpredictable step and maintaining the goal directedness of cognition across the duration of the task episode.

Conceiving a behavioral sequence as one task episode is to also conceive of a goal that the episode will achieve. Goals conceptions are considered important for the control and execution of actions that lead to their attainment (Anderson, 2014; Gollwitzer & Bargh, 2005; Greenwald, 1972; James, 1890; Jeannerod, 1988; Lewin, 1926; Meyer & Kieras, 1997; Prinz, 1987). For goals to retroactively control the episode leading to their achievement, some goal-directed cognitive entity has to actively subsume the execution of the task episode (e.g., James, 1890; Kruglanski & Kopetz, 2009). Tasks and goals, therefore, may not just exist as declarative linguistic representations, but may be better understood as hierarchical cognitive entities that subsume and control cognitive processing across the period that culminates in their completion. Attention and other control processes are always instantiated in the context of some larger task episode to achieve some goal, and hence may be instantiated through the intermediation of such goal-directed entities. We call these subsuming goal-directed cognitive entities—*task episode related programs*. (See “General discussion” for more details).

We hypothesized that any extended sequence of abstract behavior if conceived as one task episode will be executed through a subsuming program, making the corresponding cognition hierarchical. Consequently, the three signs that the previous studies have linked to the presence of hierarchical task-sequence representations will be present whenever a sequence of trials is construed as a task episode, even when those trials are not related to a memorized task-sequence representation. (1) Beginning a task episode will require assembling the program related to it, hence the high trial 1 RT. (2) Longer/more complex episodes require program of greater magnitude, hence higher trial 1 RT for longer/more complex episodes. (3) Since the program subsumes the task episode, trial-related cognitive configurations, associations, memory structures etc. will be nestled under it, a change at the higher level program at episode boundaries will necessarily change these lower level trial-related configurations, leaving no benefit/cost for repeating/switching a rule across consecutive trials that cross episode boundaries.

The presence of such episode-related programs was suggested by a neuroimaging experiment (Farooqui, Mitchell, Thompson, & Duncan, 2012). Participants were to sequentially search and detect four pre-specified letter targets while viewing a sequence of single letter presentations. Although the four target detections were identical, participants were instructed that the first three searches were part of one subtask, while the fourth one was a separate subtask. If this biasing was successful, participants would conceive the third target detection as completing a subtask episode, while the first and second target detections would be events lying within this episode that perhaps completed sub-sub-tasks within this subtask episode. The fourth target detection, on the other hand, completed the main task episode. Note that the sequential searches were identical and there was nothing structural in them that would distinguish third and fourth target detections from the first two, apart from their position in the conceived task episode. In fact, these searches could be equally well executed if these target detections were conceived as a flat sequence of four searches.

The third target detection completing the conceived subtask episode elicited greater and more widespread brain activity compared to first and second target detections that occurred within that episode. In comparison, the fourth target completing the main task episode elicited maximal activity. This pattern of elicited activity: sub-subtask completion < subtask episode completion < task episode completion, could not have resulted from changes in attention, working memory, task rules etc., because all targets were identical in these terms. The most plausible account was that these activities reflected the conceived organization of the task episode and resulted from a change in cognitive entities related to them. Completion of a lower level episode elicited limited activity, because it only dismantled the program related to it leaving that related to the higher level episode intact. Completion of the higher level episode on the other hand elicited intense and widespread activity, because it dismantled programs at both higher and lower levels of hierarchy.

### Current study

We predicted that to the extent an extended behavior is construed as one task episode it will be executed as one cognitive entity through one episode-related program, irrespective of the predictability of its components. The three signs of hierarchical cognition seen previously during the execution of memorized sequences will be seen whenever any arbitrary segment of behavior is construed as a task episode, even when mnemonic representations of the sequence are absent and the corresponding behavior is unpredictable.

Participants executed trials on which one of two items could be executed depending on the color of stimulus margins (blue: choose the lower value between the two numbers; green: choose the smaller font). The item to be executed on any trial was not known beforehand, and the probability of it being repeated or switched across successive trials was the same. Across different experiments a variety of means was used to bias subjects into conceiving a sequence of consecutive trials (or a period of time in experiment 5) as a defined task episode. These included having to additionally count trials in threes (1–2–3) or fives (1–2–3–4–5; experiments 1 and 6), temporal grouping of trials along with an irrelevant countdown (experiments 2, 3 and 4), and the presence of an irrelevant outer margin that stayed on for extended epochs of time and framed the execution of an extended series of trials (experiment 5).

If trials of such construed task episodes were executed through an episode-related program then (1) trial 1 RT will be longest because of the additional time needed to assemble the program. (2) Trial 1 RT will be longer for longer episodes, because the program related to the longer episode will take longer to assemble compared to that related to shorter episodes. (3) Trial rule related switch cost will be absent at position 1, because the dismantling and reassembly of the subsuming episode-related program at the episode boundaries will refresh lower level trial rule related configurations.

## Experiment 1

### Methods

**Fig. 1:**
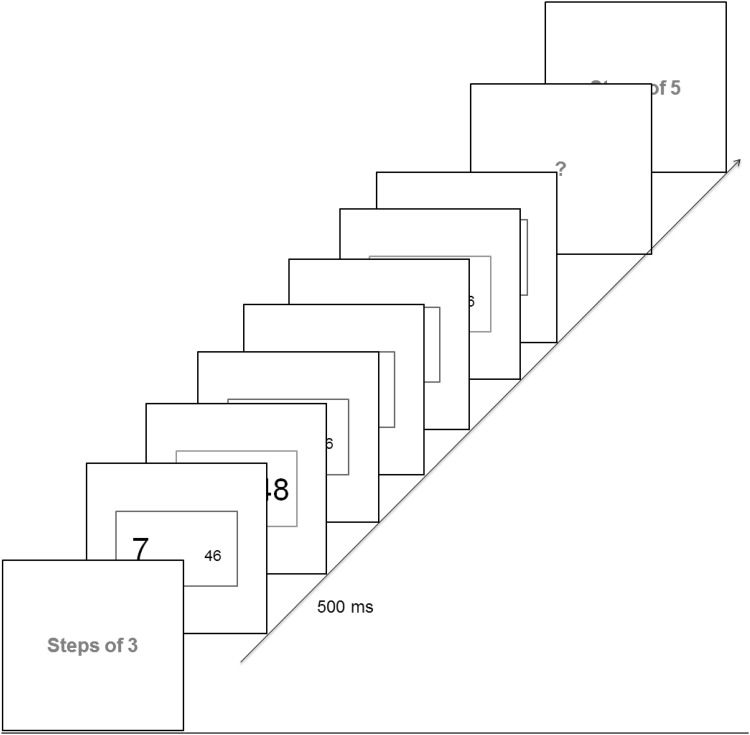
Trial blocks began with an instruction screen stating whether trials were to be counted in threes or fives. Subsequent trials were to be executed while keeping a covert count (e.g., steps of 3: 1–2–3–1–2–3…). Trial rule was cued by the color of the outer margins—blue: choose the smaller value, green: choose the smaller font. The block would end with a ‘?’, to which participants keyed in the number of the step just executed. (Color figure online)

Participants were asked to keep a covert count of trials in threes (1–2–3–1–2–3) or fives (1–2–3–4–5–1–2–3–4–5) depending on an onscreen (‘steps of 3’ or ‘steps of 5’) instruction at the beginning of each block consisting of 6–35 trials (Fig. 1). This instruction remained on until participants pressed the spacebar. On each trial two numbers differing in value (between 0 and 99) and font size (Arial font 60 or 20) were displayed on each side of the fixation cross. A margin box appeared around, and at the same time as, each number display. Margin color determined the rule relevant on that trial—blue: choose the smaller value, green: choose the smaller font. Participants’ decisions were conveyed via buttons presses that were spatially congruent with their choice (Numpad 1 for left and Numpad 2 for right on a standard QWERTY keyboard number pad). In this and all subsequent experiments the stimuli remained onscreen until a response was made. Following the response there was a fixed interval (iTi) of 500 ms. before the onset of the next stimulus/margin.

To encourage participants to view the 3- or 5-trial groups as task episodes and to check whether they were oriented to their stage within them, a probe (“?”) appeared at the end of the 6–35 trial blocks. Participants were to key in the episode step number that they had executed immediately before the probe appeared. For example, in a 3-trial episode block that ended after seven trials, the correct response would be 1 (1–2–3–1–2–3–1-probe). Feedback was given on the accuracy of probe responses (high pitch tone for correct, low pitch tone for incorrect).

This and subsequent experiments were created in Visual Basic.net and run on a Dell computer with an 85 Hz refresh rate monitor situated at a comfortable distance from the participant. The experiment was conducted on an individual basis in a testing room designed to minimize visual and noise distraction. Participants were first given seven trials of practice on each of the two trial types (value and font judgments). They then completed a 30-trial practice block in which the stimulus margin color, signaling the currently relevant rule, changed randomly. They were then told to execute trials while keeping a count in threes. They practiced on two such blocks before proceeding to the main experimental session. Participants completed 70–120 blocks each consisting of 6–35 trials with 3- and 5-trial blocks being randomly interlaced. Participants were to respond as quickly and as accurately as possible.

#### Participants

Fifteen healthy participants (nine females) were recruited through MRC-CBU volunteers’ panel. Their age group in this and in subsequent experiments was 18–40. All gave written, informed consent before the experiment, and were paid £8.50 for their participation. All had normal or corrected to normal vision.

### Results

**Fig. 2:**
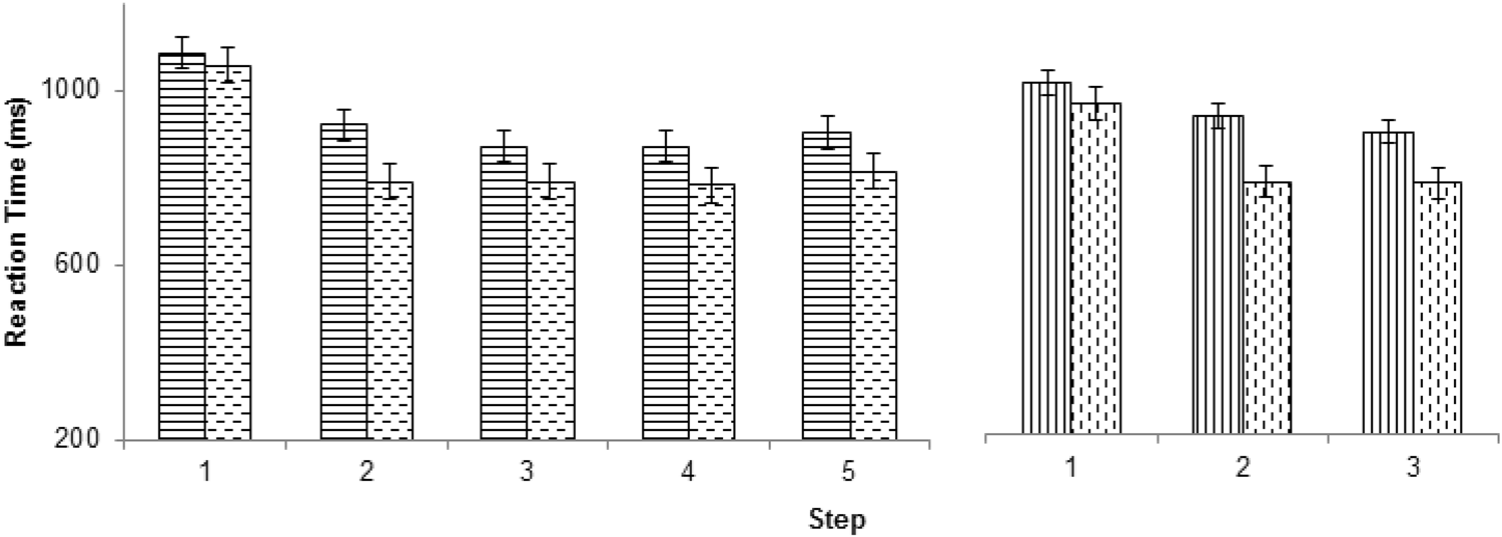
Pattern of reaction times across the steps of the 5- and 3-trial episodes (continuous line bars: rule switch trials, dashed line bars: rule repeat trials). Note that trial 1 had the highest RT for both switch and repeat trials in both 5- and 3-trial episodes. Error bars represent 95% confidence intervals calculated using the method suggested by (Loftus & Masson, 1994) for within subject analyses

Figure 2 and Tables 1 and 2 summarize the main results. As is evident, these concur with the key predictions: (1) significantly elevated RTs to the first trial of each construed episode; (2) longer RTs for 5-trial than 3-trial episodes; and (3) lack of significant switch costs when switches occurred across the boundaries between episodes. The first trial of the conceived episode took longest to execute (Table 1, and main effect of serial position in Table 2). Cohen’s *d* (effect size: mean difference/standard deviation) was 1.68 and 1.99 for 3 and 5-trial episodes, respectively. This trial 1 RT was higher for 5-trial compared with 3-trial episodes [95% CI of difference = (46, 113), Table 1 and interaction between the effects of serial position and episode length in Table 2, Cohen’s *d* = 1.3]. While the performance on switch trials was poorer than on repeat trials (Table 2, main effect of rule switch), this effect of rule switch differed across the serial positions within the episode (Table 2: Rule Switch × Serial Position). Specifically, as predicted no statistically significant switch costs were observed at trial 1 [RT: paired *t*_14_ = 1.05, *p* = 0.3, 95% CI of difference = (− 19, 54); accuracy: paired *t*_14_ = 1.1, *p* = 0.3, 95% CI of difference = (− 0.02, 0.01)].

**Table 1:** Mean reaction times (ms) and accuracies (%, bottom of each cell) along with their 95% confidence intervals across rule switch and repeat trials of 3- and 5-trial episodes

	3-trial episodes			5-trial episodes				
	1	2	3	1	2	3	4	5
Switch	1014 ± 27 93 ± 1.1	938 ± 18 93 ± 0.9	902 ± 16 93 ± 1.0	1084 ± 42 95 ± 1.0	920 ± 18 93 ± 1.0	871 ± 17 94 ± 1.2	870 ± 27 93 ± 1.7	903 ± 20 94 ± 1.1
Repeat	968 ± 31 94 ± 0.8	785 ± 18 95 ± 0.5	782 ± 20 96 ± 0.9	1058 ± 50 95 ± 1.1	792 ± 19 96 ± 1.2	791 ± 28 95 ± 1.2	783 ± 19 94 ± 1.4	814 ± 23 97 ± 1.3

**Table 2:** Serial position: repeated measures ANOVA looking at the main effect of the position of trial within the episode on RT (and, in parenthesis, accuracy)

Effect	*df*s	*F*	MSE	*p*
Serial position (three step)	2,28	37.9 (1.55)	196,781 (0.001)	< 0.001 (0.23)
Rule switch (three step)	1,14	32.3 (9.86)	255,789 (0.11)	< 0.001 (< 0.01)
Rule switch × serial position (three step)	2,28	18.7 (0.52)	22,258 (0.001)	< 0.001 (0.6)
Serial position (five step)	4,56	46.9 (3.07)	317,819 (0.002)	< 0.001 (0.02)
Rule switch (five step)	1,14	26.0 (12.4)	256,207 (0.012)	< 0.001 (< 0.01)
Rule switch × serial position (five step)	4,56	5.7 (1.76)	9956 (0.002)	0.001 (0.15)
Episode length	1,14	2.6 (0.5)	20,059 (0)	0.13 (0.5)
Serial position × episode length	2,28	24.2 (1.5)	39,053 (0.001)	< 0.001 (0.25)

Rule switch: main effect of rule switch (switch vs repeat trials). Rule switch × serial position: interaction between the effects of rule switch and serial position. Serial position × episode length: effect of serial position compared across three step and the first three trials of five step episodes

It’s noteworthy that while the elevated RTs on switch trials compared to repeat trials were accompanied by the expected reduced accuracy, the elevated RTs on trial 1 compared with within-episode trials was, if anything, associated with greater accuracy. This was the case even though the within-episode switch cost was 92 ms, while the episode onset ‘cost’ (relative to within-episode trials) was in the region of 150–200 ms. This is to be expected, because response on switch trials got delayed because of control demands that was intrinsic to executing those trials, e.g., choosing the correct response in light of the newly relevant rule and overcoming interference from associations and configurations related to the previous rule. In contrast, trial 1 response got delayed because the episode-related program had to be assembled prior to the actual execution of trial 1, control demands related to choosing the correct response of trial 1 remained the same.

## Experiment 2

In experiment 2, we replicated the basic findings of Experiment 1 using a different design and tested an additional prediction. If trials of a task episode are executed through one program assembled at trial 1 then later trials of the episode, i.e., trial 2 onwards, may be executed faster than identical trials executed as independent tasks. This is because when trials are executed as independent tasks they will have to be individually prepared for and the related program will have to be individually assembled before every trial. In contrast, when trials are executed as parts of a task episode they are prepared for as one unit and the related common program embodying these preparations assembled at trial 1. These preparations will not be individually made prior to every trial, resulting in the faster execution of later trials.

**Fig. 3:**
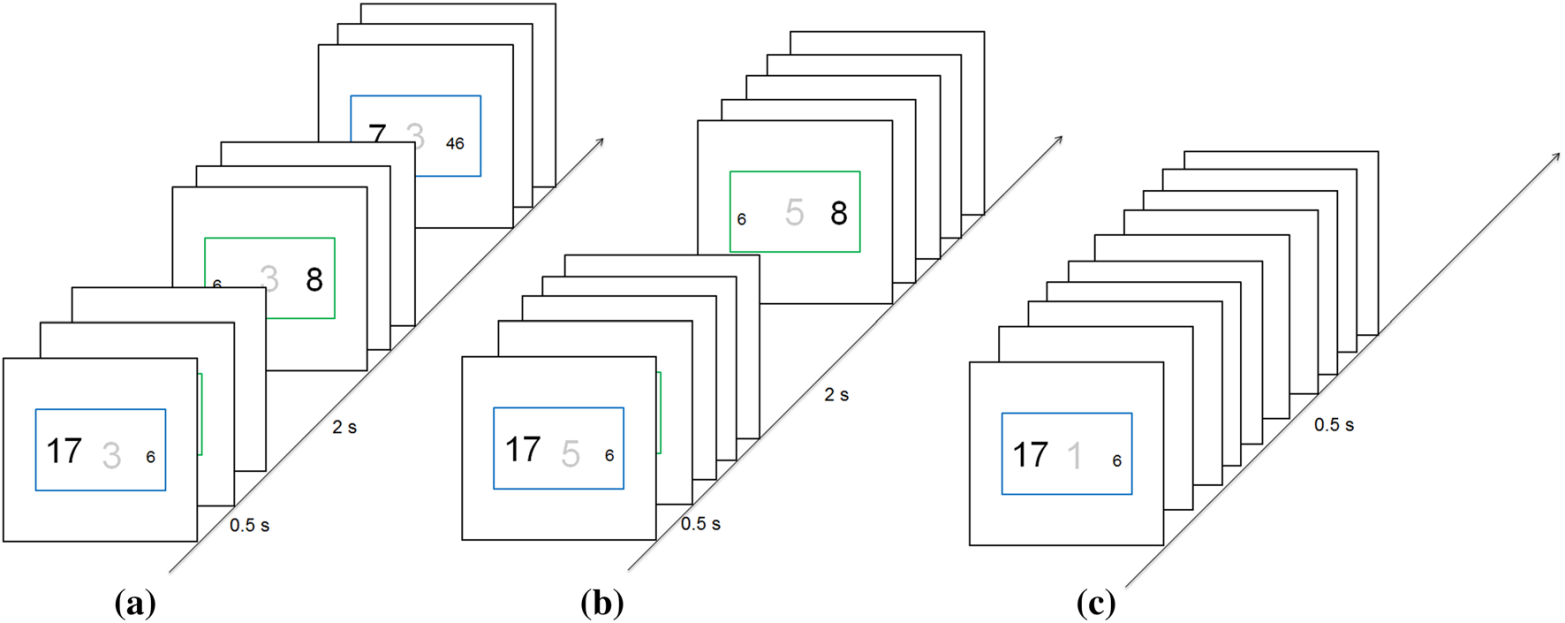
Trial rules were the same as in Experiment 1. There were three kinds of trial blocks. **a** 3-trial episodes were created by having small iTi within the episode and large iTi across episode boundaries. This was further reinforced by a faded digit in the background that went from 3 to 1 across the 3 trials making up the episode. **b** 5-trial episodes were similar to the 3 trial episodes, except that they consisted of 5 trials, and the faded background digit went from 5 to 1 across them. **c** Trials of the independent trial blocks were presented in a flat sequence with constant iTi and had the digit ‘1’ in their background that remained the same throughout the block. (Color figure online)

We used a different method to bias participants’ conception of what constituted a task episode. Series of 3 or 5 consecutive trials were grouped together by having smaller inter-trial intervals (500 ms, iTi) between them, while these episodes were separated from each other by discernibly larger durations (2 s; Fig. 3). This was further reinforced by a faded number in the stimulus background that conveyed the number of steps left in the current episode. The first trial of a 3-trial episode had the digit ‘3’ in the background, while the second trial had ‘2’ and the third had ‘1’ (Fig. 3a). Likewise, in 5-trial episodes this background digit changed 5-4-3-2-1, across its 5 trials (Fig. 3b). Note that trials (and by extension the experiment) could be executed perfectly well without construing the series of trials as a task episode. There was nothing structural in the design that forced participants to construe the 3 (or 5) consecutive trials as parts of a larger task episode. Apart from blocks composed of 3- and 5-trial episodes there was a third block type whose trials were not organized into episodes and were instead presented as one flat sequence with digit 1 in the stimulus background (Fig. 3c).

### Methods

Stimuli and trial rules were identical to experiment 1 (Fig. 3). Before 3- and 5-trial task episode blocks participants saw ‘3 Step Tasks’ and ‘5 Step Tasks’, respectively, this instruction screen remained till the spacebar was pressed. Before the independent trial block participants saw ‘Independent Trials’, and the individual trials of this block had the number ‘1’ in the stimulus background. The 3- and 5-trial task blocks had 70 and 120 trials, respectively, and the independent trial blocks had 70 trials. The order of the three block types was random. Eighteen participants (11 females) did a total of 85–100 blocks. They were not explicitly told about the temporal grouping of the trials in the main experiment, and were asked to ignore the faded number in the stimulus background.

### Results

Tables 3 and 4 summarize the key results. The first trial of the 3- and 5-trial episodes took longest to execute (Cohen’s *d* 1.3 and 1.6; Table 3, and main effect of serial position in Table 4). While there was a main effect of rule switch on performance (main effect of rule switch in Table 4), this varied across serial positions (Table 4: rule switch × serial position). Specifically, as in the previous experiment, it was not significant on trial 1 [RT: paired *t*_17_ = 1.7, *p* = 0.1, 95% CI of difference = (− 8, 71); accuracy: paired *t*_17_ = 0.8, *p* = 0.4, 95% CI of difference = (− 0.01, 0.02)]. Unlike the previous experiment, the trial 1 RT for 5-trial episodes was not higher than 3-trial episodes [95% CI of difference = (− 39, 59); Table 4 interaction between serial position and episode length, Cohen’s *d* = 0.1]. Finally, as in the previous experiment, the strong difference in RT between the first and subsequent steps was not accompanied by changes in accuracy (Table 3, and main effect of serial position on accuracy in Table 4).

**Table 3:** Mean reaction times (ms) and accuracies (%, bottom of each cell) along with their 95% confidence intervals across rule switch and repeat trials of 3- and 5-trial episodes as well as those from the independent trial blocks

	3-trial episodes			5-trial episodes					Independent trials
	1	2	3	1	2	3	4	5	
Sw	1052 ± 33 96 ± 0.7	950 ± 17 96 ± 1.2	905 ± 32 94 ± 1.3	1048 ± 40 95 ± 0.9	951 ± 18 96 ± 1.5	940 ± 13 94 ± 1.2	979 ± 28 94 ± 1.7	952 ± 26 94 ± 1.1	1052 ± 43 95 ± 1.1
Rp	1007 ± 55 95 ± 1.3	748 ± 36 95 ± 1.0	769 ± 23 96 ± 1.2	1030 ± 46 95 ± 1.1	773 ± 20 97 ± 1.0	782 ± 20 96 ± 1.2	814 ± 15 96 ± 0.7	822 ± 25 96 ± 1.4	875 ± 40 98 ± 1.1

**Table 4:** Serial position: repeated measures ANOVA looking at the main effect of the position of trial within the episode on RT (and accuracy)

Effect	*df*s	*F*	MSE	*p*
Serial position (three trial)	2,34	29.6 (0.44)	419,633 (0.001)	< 0.001 (0.6)
Rule switch (three trial)	1,17	43.2 (0.9)	440,396 (0.001)	< 0.001 (0.3)
Rule switch × serial position (three trial)	2,34	14.2 (4.02)	55,634 (0.003)	< 0.001 (0.02)
Serial position (five trial)	4,68	31.5 (1.3)	199,388 (0.001)	< 0.001 (0.3)
Rule switch (five trial)	1,17	39.5 (9.4)	758,310 (0.01)	< 0.001 (< 0.01)
Rule switch × serial position (five trial)	4,68	13.9 (1.4)	37,673 (0.001)	< 0.001 (0.2)
Episode length	1,17	1.3 (0.02)	13,394 (0)	0.3 (0.9)
Serial position × episode length	2,34	0.324 (1.5)	1013 (0.001)	0.7 (0.25)

Rule switch: main effect of rule switch on RT (and accuracy). Rule switch × serial position: interaction between the effects of rule switch and serial position. Serial position × episode length: effect of serial position compared across 3-trial and the first three trials of 5-trial episodes

Trials conceived as parts of a task episode were executed faster than independent trials. As is evident from Table 3 the switch and repeat independent trials were higher than RTs on trials 2 and beyond of task episodes (*t*_17_ > 5.5, *p* < 0.001; Cohen’s *d* > 1.61). Accuracies, however, were not significantly different. Thus, organization of trials into larger task episodes created a slower trial 1 but resulted in faster execution of subsequent trials. Was the price paid at trial 1 offset by gains at subsequent steps? We compared average RT and accuracy between blocks organized into task episodes with those that were not. Average RT on 3- and 5-trial task blocks was still significantly lower than on the independent trial blocks (*t*_17_ > 4.1, *p* < 0.001, Cohen’s *d* = 0.82). This benefit also extended to switch control. Average reaction time switch cost within independent trials was greater than that amongst trials executed as parts of task episodes [176 vs 128 ms; *t*_17_ = 2.1, *p* = 0.06, CI of difference = (− 1, 96), Cohen’s *d* = 0.49].

Improved performance within task episode trials could have been due to the participants becoming more fatigued during independent trial blocks due to the absence of slightly longer inter-trial intervals that marked task episode boundaries. To examine this we compared the initial 30 trials of the independent trial blocks with trials of the latter half of 3- and 5-trial episode blocks. Participants may be expected to be less tired during the former (as they had only executed a maximum of 30 trials) than the latter (where they would have executed between 35 and 60 trials in 3- and 5-trial episodes, respectively). However, results were identical to the above and participants were still faster during task episode (*p* < 0.01, Cohen’s *d* = 0.87) compared to independent trials.

Another possibility may be that during independent trial blocks participants were executing an extended continuous sequence, which may have resulted in attentional breaks. Trials following such breaks can be expected to have very high RTs. In contrast, while executing task episode participants got long temporal breaks after every 3 and 5 trials during episode blocks that could have refreshed and refocused their attention before the next bout of 3 or 5 trials. As per this account the more frequent presence of post-attentional break high RT trials in the continuous trial blocks may have increased their average RTs compared to task episode blocks. This explanation predicts that the RT distribution of independent trials should primarily differ from that of task episode trials in having an additional bump made of these high RT post-attentional break trials in the right sided tail of the distribution. Otherwise, other aspects of their RT distributions, e.g., the mode of their distributions (most frequent RT values), should be identical.

**Fig. 4:**
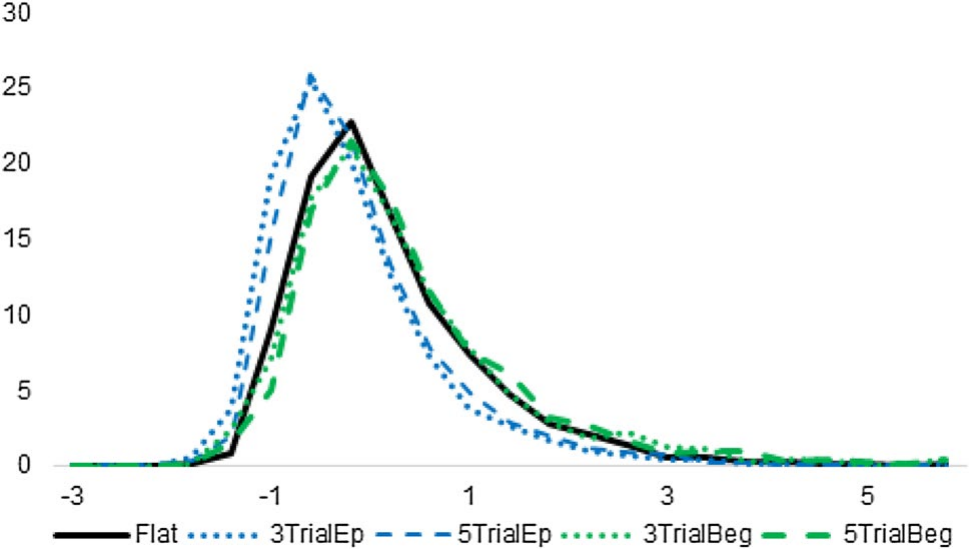
Distribution of *z*-scores of RTs of trials from independent trial blocks (black line), trial 2 and beyond of 3- and 5-trial episodes (blue lines) and trial 1 of 3 and 5 trial episodes (green lines). *y*-axis represents the percentage of trials having a particular *z*-score. While more frequent attentional breaks during independent trial blocks would have predicted that the black and blue lines differ mainly in their tails (see text), in reality these lines differed in their entire distribution and not just in their tails. (Color figure online)

To plot RT distributions we first converted individual RTs to *z*-scores. We categorized all trials into four categories on the basis of the rule executed (value or font) and whether these rules were switch or repeat from the previous trial. The difference of every RT from the mean of its category was divided by the standard deviation of that category to get a *z*-score (zRT) corresponding to that RT. The blue lines in Fig. 4 show the distribution of zRTs from trials 2 and beyond of 3- (dotted blue) and 5-trial (dashed blue) episodes. The black line shows the distribution from the independent trial block. Note that while the zRT distributions of trials from 3- and 5-trial episodes are largely congruent, that of the independent trials is very different. Crucially, this difference is not limited to an additional bump in the tail of the distribution (corresponding to the purported post-attentional break very long RT trials), instead the entire distribution of independent trial zRTs is shifted to the right compared to the zRTs from within task episodes. In fact the distribution of zRTs from the independent trial blocks was largely congruent with those of trial 1 of 3- and 5-trial episodes (green plots). Hence, the above observation that trials (subsequent to trial 1), construed as parts of a task episode, were executed faster than identical trials, executed as independent entities, cannot be attributed to more frequent lapses in sustained attention during the independent trial blocks.

## Experiment 3

Experiment 2 replicated two out of the three main results of experiment 1. The exception was the absence of a significant difference in RT at the beginning of 3- compared with 5-trial task episodes. One possibility is that this arose because each episode type was blocked, meaning that preparation for a task episode became somewhat routine. We examined this in experiment 3 using an identical design but now randomly interleaving 3- and 5-trial task episodes in the same blocks.

### Methods

The methods here were identical to Experiment 2 with the exceptions that 3- and 5-trial episodes occurred randomly within the same block and there was no continuous block condition. The experimental session lasted half an hour during which participants did an average of 250 task episodes, which consisted of roughly equal number of 3- and 5-trial episodes. Twenty-two healthy participants (11 females) participated.

### Results

Tables 5 and 6 summarize the key results. Notably, the trial 1 RT was higher for 5-trial compared to 3-trial episodes [Tables 5 and 6, interaction between serial position and episode length, 95% CI of difference = (15, 96), Cohen’s *d* = 0.6]. Other results were largely a replication of the previous two experiments. The first step of the conceived episode took longest to execute (Table 5, and main effect of serial position in Table 6). This was the case for both switch and repeat trials at trial 1. Performance on switch trials was poorer than on repeat trials (Table 6 main effect of rule switch); however, the effect of rule switch was not the same across the trials making up the episode (Table 6: rule switch × serial position). Specifically, the switch cost was not significant at the first position [RT: paired *t*_21_ = 0.89, *p* = 0.4, 95% CI of difference = (− 43, 109); accuracy: paired *t*_21_ = 0.3, *p* = 0.7, 95% CI of difference = (− 1.4, 1.9)]. Finally, accuracies were not significantly different between the trial 1 and subsequent trials (main effect of serial position on accuracy in Table 6).

**Table 5:** Mean reaction times (ms) and accuracies (%, bottom of each cell) along with their 95% confidence intervals across rule switch and repeat trials of 3- and 5-trial episodes

	3-trial episodes			5-trial episodes				
	1	2	3	1	2	3	4	5
Sw	1275 ± 68 97 ± 0.9	1125 ± 38 97 ± 0.7	1116 ± 34 97 ± 0.9	1333 ± 85 97 ± 0.7	1108 ± 28 97 ± 0.6	1112 ± 39 97 ± 0.7	1158 ± 27 97 ± 0.7	1154 ± 37 97 ± 0.8
Rp	1248 ± 73 96 ± 0.9	925 ± 45 99 ± 0.6	947 ± 38 98 ± 0.6	1300 ± 85 96 ± 1.0	904 ± 33 98 ± 0.7	934 ± 33 98 ± 0.6	977 ± 28 98 ± 0.6	990 ± 27 97 ± 0.6

**Table 6:** Serial position: repeated measures ANOVA looking at the main effect of the position of trial within the episode on RT (and accuracy)

Effect	*df*s	*F*	MSE	*p*
Serial position (three step)	2,42	20 (2.9)	797,993 (14.1)	< 0.001 (0.06)
Rule switch (three step)	1,21	39.7 (2.2)	577,148 (27.1)	< 0.001 (0.15)
Rule switch × serial position (three step)	2,42	11.1 (2.3)	93,740 (13)	< 0.001 (0.11)
Serial position (five step)	4,84	23.5 (2.5)	697,698 (9.9)	< 0.001 (0.05)
Rule switch (five step)	1,21	43.1 (6.2)	1,273,614 (48.8)	< 0.001 (0.02)
Rule switch × serial position (five step)	4,84	12.4 (1.9)	51,311 (7)	< 0.001 (0.12)
Episode length	1,21	0.6 (0.6)	5631 (0)	0.5 (0.8)
Serial position × episode length	2,42	11.1 (0.09)	35,336 (0.39)	< 0.001 (0.9)

Rule switch: main effect of rule switch (switch vs repeat trials). Rule switch × serial position: interaction between the effects of rule switch and serial position. Serial position × episode length: effect of serial position compared across 3-trial and the first three trials of 5-trial episodes

## Experiment 4

Switch cost is caused by a change in implementation strategies (e.g., change in task set, recall of new rules, over-riding past task-related associations/configurations etc.) between previous and the current acts that are conceived as different tasks (Kiesel et al., 2010; Rogers & Monsell, 1995). In experiments 1–3 a sequence of task items were executed as parts of a larger task episode. Cognitive accompaniments related to individual task items were subsumed by the program related to the overarching episode whose dismantling/reassembly at episode boundaries left no advantage of repeating (or cost of switching) a task item. In contrast to switch cost the congruency effect in Stroop tasks (e.g., identify the font color of the word RED when the font is blue) follows largely from the incongruence between different stimulus dimension (e.g., word meaning and color) of the current trial (MacLeod, 1991; Stroop, 1935). Sequence effects on congruency (e.g., slower response on incongruent trials following congruent trials compared to incongruent trials following incongruent trials) are present but account for only a fraction of incongruence effects (Egner, 2007). If the absence of significant switch cost at trial 1 was related to the subsuming of individual trial-related cognitive configurations by the episode-related program, then Stroop congruence effect (hence called Stroop cost) should not be absent on this trial, because these largely arise from the incongruence between the different stimulus dimensions of the same trial, and hence should not be affected by change in the subsuming program at episode boundaries.

Other accounts of hierarchy in cognition may predict a decrease in trial 1 Stroop cost. Koechlin, Ody, & Kounelher, (2003) have suggested a hierarchy of control processes, whereby (e.g.) episodic control determining what all control processes are to be instantiated in a given period subsumes the instantiation of trial rule-related control. Furthermore, these processes have a cascading relationship, e.g., the episodic control released by more anterior prefrontal regions goes through regions instantiating trial rule-related control and enhances the instantiation of this lower level control process. Such an account may suggest that the absence of trial 1 switch cost in the previous experiments was a result of the instantiation of episode-level control enhancing the instantiation of lower trial-level control (see also Schneider & Logan, 2006). This will predict that Stroop costs, another lower trial-level control demand, may also be decreased at the beginning of the episode.

It is also possible that Stroop cost be increased at trial 1. If the longer trial 1 RT was caused by a competition between the simultaneously active task episode related and trial 1 related processes for limited resources then more demanding trial 1 executions like the incongruent Stroop trials will get additionally delayed. In contrast, in our account trial 1 RT got delayed because the episode-related program had to be assembled prior to the execution of trial 1, and trial 1 like other trials was executed through this program. The processes assembling it were not in competition with those involved in processing demands intrinsic to trial 1. Consequently, our account predicts that the trial 1 Stroop cost will not only be present, it will be of the *same* magnitude as on subsequent trials.

Participants did a modification of color-word Stroop task (Fig. 6). The experiment had two kinds of blocks. Trials of the first one were temporally grouped into task episodes similar to that in Experiments 2 and 3 but in this case comprising four trials. To further encourage construal of these 4-trials as task episodes, a faded background digit counted down to the completion of each episode (4-3-2-1). In the second block type, trials were not organized into short task episodes and were presented as a flat sequence (iTi = 500 ms) with the digit ‘1’ in the stimulus background. These blocks allowed us to replicate the results of experiment 2 that had shown faster execution of trials executed as parts of a larger task episode than independent/continuous trials.

### Methods

**Fig. 5:**
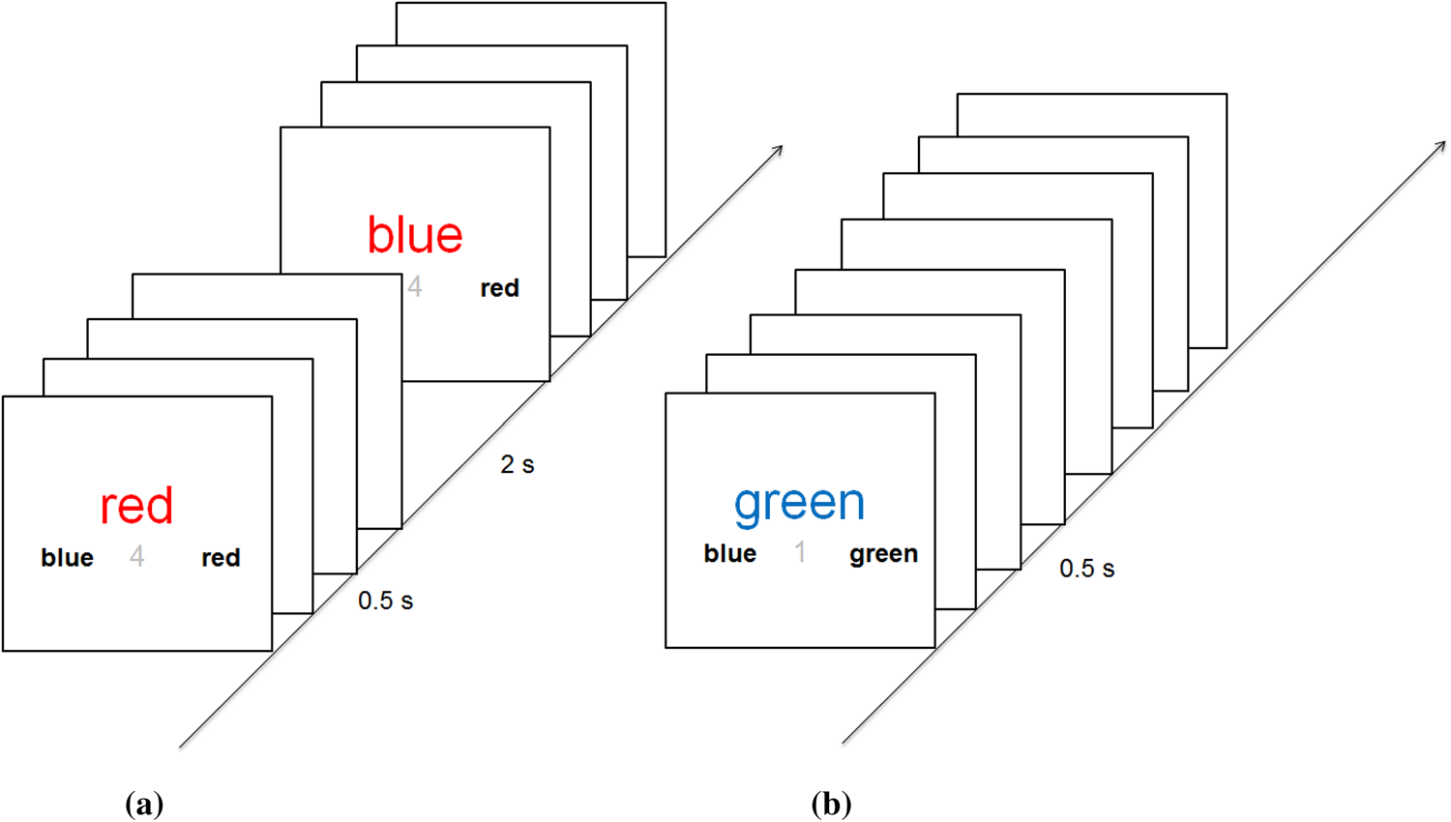
Participants indicated the color of the print of the central word by pressing the button spatially congruent with one of the two color choices presented at the bottom of the screen. They were encouraged to construe groups of four trials as a single-task episode via an extended iTi at the end of each episode and by a background faded digit counting down the sequence to its completion (4-3-2-1). In a second block type trials were presented as a flat sequence with constant iTi across the block. All trials of such blocks had the digit ‘1’ in their background. (Color figure online)

On each trial participants saw a centrally presented color word (‘Red’, ‘Blue’, ‘Green’, and ‘White’; in Arial font size 40). The word appeared in congruent font color on 60% of trials and in an incongruent color on the remainder (Fig. 5). Participants chose the color of the print from the two color words presented in Arial black font size 20 below (1 degree away from the center and ½ degree below it) by pressing a button spatially congruent with their choice - left: Numpad 1 (right index finger); right: Numpad 2 (right middle finger). One of the two bottom color words (allocated to left or right at random) always reflected the font color. When the font and word were incongruous, the other word always repeated the stimulus word. On congruous trials the other word was selected randomly from the remaining colors. Between these two options a partially (70%) transparent digit appeared in black. The stimuli remained on screen till a response was made. Erroneous responses elicited a low-pitched feedback tone.

At the start of 4-trial blocks the instruction screen mentioned ‘4—step blocks’, it remained on until the spacebar was pressed. Such blocks consisted of 96 trials. The instruction for the independent trial block mentioned ‘independent trials’. Such blocks consisted of 48 trials. These two blocks were interleaved. Eighteen participants (9 female) each completed 12 blocks.

### Results

Tables 7 and 8 summarize the key results. As before, the first trial of the episode took longest to execute [Table 7, and main effect of serial position in Table 8, 95% CI of difference = (54, 146), Cohen’s *d* = 1.1]. This was the case for both congruent and incongruent trial 1. Expectedly, the performance on incongruent trials was poorer than on congruent trials (Table 8 main effect of Stroop cost), but crucially it did not vary across trials of the episode (Table 8: stroop cost × serial position). Specifically, unlike switch cost in the previous experiments, the cost of incongruence did not disappear on trial 1 of the episode [RT: one-sample *t*_17_ = 8.8, *p* = < 0.001, 95% CI of difference = (127, 206); accuracy: one-sample *t*_17_ = 4.2, *p* ≤ 0.001, 95% CI of difference = (1.7, 5.0)].

**Table 7:** Mean reaction times (ms) and accuracies (%) across the incongruent and congruent trials corresponding to the four steps of task episode and of independent trial blocks

	1	2	3	4	Independent trials
Incongruent	1104 ± 43 96 ± 1.4	991 ± 28 94 ± 1.1	995 ± 25 94 ± 1.1	986 ± 28 94 ± 1.3	1141 ± 38 92 ± 1.4
Congruent	938 ± 30 99 ± 0.4	843 ± 20 99 ± 0.3	862 ± 19 99 ± 0.2	848 ± 23 99 ± 0.3	1000 ± 38 99 ± 0.4

**Table 8:** Serial Position: Repeated measures ANOVA looking at the main effect of the position of trial within the episode on RT (and accuracy, in parenthesis)

Effect	*df*s	*F*	MSE	*p*
Serial position (four step)	3,51	18.3 (1.8)	90,838 (1821)	< 0.001 (0.2)
Stroop cost (four step)	1,17	92.1 (87.9)	769,757 (726,102)	< 0.001 (0.3)
Stroop cost × serial position (four step)	3,51	2.1 (2.4)	1965 (2115)	0.1 (0.08)

Stroop cost: main effect of congruence (incongruent vs congruent trials). Stroop cost × serial position: interaction between the effects of congruence and serial position

RTs on incongruent and congruent trials of the independent trial blocks were higher than the incongruent and congruent trials of task episodes. The average RT on trials executed as parts of task episodes was lower than for independent trials even when RTs of trial 1 of task episodes were included in comparison [congruent trials: *t*_17_ = 7, *p* < 0.001, 95% CI of difference = (89, 165); incongruent trials: *t*_17_ = 4.9, *p* < 0.001, 95% CI of difference = (69, 175)]. While there was a general RT advantage for task episode trials, Stroop cost on RT (RT on incongruent trials − RT on congruent trials) did not differ between task episode and independent trials [*t*_17_ = 0.3, *p* = 0.8, 95% CI of difference = (− 27, 37)]. However, Stroop cost on accuracy (accuracy on Congruent trials − accuracy on incongruent trials) was significantly higher on independent compared to task episode trials [*t*_17_ = 2.6, *p* = 0.02, 95% CI of difference = (0.5, 5.5)].

The results affirmed the general pattern of the previous results in showing elevated RTs on the first trial of episodes in the context Stroop task. They confirmed the prediction that Stroop cost would be present on trial 1 and have the same value as on other trials of the episode. They also replicated the findings of experiment 2 that trials executed as parts of a task episode got executed faster and better controlled than trials executed as independent tasks.

## Experiment 5

Counting or countdowns involved in prior experiments could be construed as hierarchical acts, and it is possible that the hierarchy evident in the execution of trials in experiments 1–4 was a spillover from a separate but simultaneous hierarchical task—counting (or countdown) in 3, 5 or 4s. Through this experiment we clarify that this was not the case, the above observations resulted from the construal of the trial series as one task episode.

**Fig. 6:**
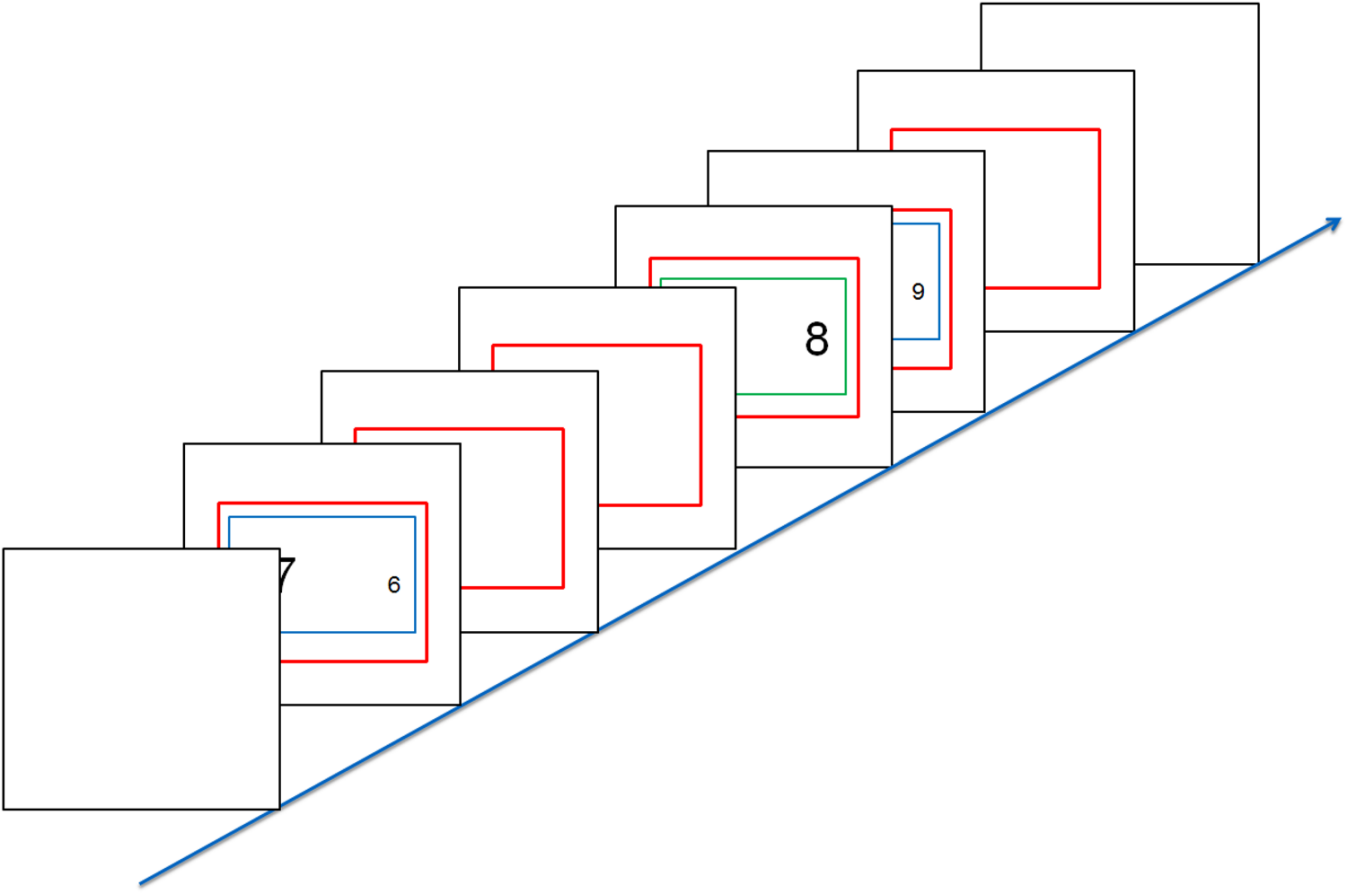
Outermost margin (here in red) remained onscreen for an extended duration during which an unpredictable number of trials would appear at random intervals. Trials would not appear when this margin was off. We hoped that participants would construe the temporal epochs carved by the presence of such margins as the recurring task episodic units to be executed. (Color figure online)

The task episodes of the current experiment did not have a fixed number of trials and did not involve counting or countdown. Instead the notion of episodes was created by the presence of an additional margin (outside the one conveying the relevant trial rule) that stayed on for an extended duration during which trials would appear randomly at any time. Trials would not appear after this margin had switched off. Every episode began with the appearance of trial 1 stimulus along with this margin (Fig. 6). While the trial 1 stimuli disappeared after the response, the outer margin remained on, and subsequent trials would appear anytime, while this outer margin was on. This margin went off at the end of the episode, and came back on with the beginning of the next episode. The notion of task episode was thus implicitly conveyed as the period enveloped by the presence of the outer margin. Participants were not instructed about the episodic structure of the task block and were told nothing about the outermost margins.

Two kinds of episodes (short and long), framed by different color margins (black or red), were randomly interleaved. Short episodes lasted between 3 and 6 s during which two to three trials could appear. Long episodes lasted between 7 and 10 s during which three to seven trials could appear. The inter-episode interval was fixed at 2 s, but the iTi within an episode varied from 50 ms to 5 s in short episodes and 50 ms to 3 s in long episodes. Note that in this experiment, not only did the subjects not have any foreknowledge about the identity and sequence of task items, they also could not predict the number of trials and their iTis. In addition, note that this design created greater uncertainty about the timing of later trials compared to trial 1. While the timing of trial 1 was fixed at 2 s after the last offset of the outer (episode-related) margin, subsequent trials could occur anywhere from 50 ms to 5 s after the previous trial. This uncertainty is likely to slower the RT of later trials, especially if preceded by very long iTis (Gopher, Armony, & Greenshpan, 2000). The presence of signs of task episodes in the face of the very implicit nature of current task episodes and the uncertainty associated with individual trials would testify to the pervasiveness and strength of the cognitive tendency to recognize and utilize the episodic organization of the task at hand for the execution of behavior.

### Methods

Apart from the additional outer margin, individual trials were identical to Experiments 1–3 (speeded decision as to which of the two numbers had the smaller value or font size as indicated by the color of the inner margin). 27 participants (17 females) did two experimental sessions each lasting 16 min, with a period of rest in between. Within a session the two kinds of conceived episodes occurred randomly.

### Results

Key results of the previous experiments were again replicated (Tables 9, 10). The first trial of the episode took longest to execute (*F*_2,52_ = 28.1, *p* < 0.001). This trial 1 RT was longer before longer episodes [95% CI of difference = (14, 39), Cohen’s *d* = 0.81; *t*_26_ = 4.2, *p* < 0.001]. Switch cost was substantially reduced at the first trial of the episode (Smaller episodes: *F*_2,52_ = 18; Longer episodes: *F*_6,156_ = 8.1, *p* < 0.001 for both). The execution of trials construed as parts of a larger task episode again showed key behavioral signatures that suggested that these trials were executed not as independent entities but through a common program. This was the case even though participants were unaware of the number and sequence of task items they would execute as well as when those items would be executed and the precise duration of the episode.

**Table 9:** Mean reaction times (ms) and accuracies (%) across the switch and repeat trials of short and long episodes

	Short			Long						
	1	2	3	1	2	3	4	5	6	7
Sw	873 ± 16 94 ± 1.5	876 ± 11 94 ± 1.3	876 ± 14 92 ± 1.2	898 ± 24 92 ± 16	853 ± 17 92 ± 16	883 ± 19 94 ± 16	867 ± 21 94 ± 16	874 ± 16 92 ± 16	851 ± 17 93 ± 16	886 ± 19 92 ± 16
Rp	843 ± 24 95 ± 1.0	723 ± 16 97 ± 1.0	751 ± 22 97 ± 0.9	856 ± 18 94 ± 1.2	723 ± 15 96 ± 2	722 ± 17 97 ± 1.2	741 ± 13 95 ± 1.8	755 ± 15 95 ± 2.2	775 ± 13 95 ± 1.7	746 ± 10 97 ± 1.5

**Table 10:** Serial position: repeated measures ANOVA looking at the main effect of the position of trial within the episode on RT (and accuracy, in parenthesis)

Effect	*df*s	*F*	MSE	*p*
Serial position (short)	2,52	28 (1.2)	163,851 (0.002)	< 0.001 (0.3)
Rule switch (short)	1,26	119 (17.9)	544,179 (0.05)	< 0.001 (< 0.001)
Rule switch × serial position (short)	2,52	18 (4.7)	51,458 (0.007)	< 0.001 (0.01)
Serial position (long)	6,156	11 (2)	38,664 (0.004)	< 0.001 (0.07)
Rule switch (long)	1,26	135 (16.2)	1,243,930 (0.09)	< 0.001 (< 0.001)
Rule switch × serial position (long)	6,156	8.1 (1.4)	19,205 (0.002)	< 0.001 (0.2)
Episode length	1,26	0.02 (1.3)	87 (0.003)	0.9 (0.3)
Serial position × episode length	2,52	6.1 (0.07)	3840 (0.4)	< 0.01 (0.9)

Rule switch: main effect of rule switch (switch vs repeat trials). Rule switch × serial position: interaction between the effects of rule switch and serial position. Serial position × episode length: effect of serial position compared across short and the first three trials of long episodes

Note that the trial 1 RT in this experiment was delayed by only 64 ms compared to subsequent trial RTs. In comparison, in experiments 1 to 3 this was 175–250 ms. This is likely to be due to greater uncertainty related to the timings of later trials compared to trial 1. While trial 1 would predictably occur 2 s from the offset of the previous task episode margin, subsequent trials would appear variably between 50 ms to 5 s after the previous trial. This can be expected to decrease trial 1 RT while increasing RTs on subsequent trials (Table 9).

## Experiment 6

In this experiment, we investigated two questions. First, is the higher trial 1 RT during 5-trial episodes compared to 3-trial episodes caused by their greater number of trials or their greater temporal duration? Second, is the difference in RTs between longer and shorter episodes limited to trial 1? Through the first question we investigated if the magnitude of the subsuming program is also related to the temporal duration of the episode, and through the second, whether maintaining the program impinged upon the limited cognitive reserves available for trial execution.

The first question could not be answered by the previous experiments because episodes longer in duration also had greater number of trials. To the second question experiments 1–5 gave a null result. While trial 1 RT was longer before longer episodes, RTs on subsequent trials were not different between longer and shorter episodes. Hence in all these experiments only the interaction between the effects of serial position with episode length was significant but the main effect of episode length was not (Tables 2, 4, 6, 10). Maintaining the larger program did not seem decrease the availability of limited capacity reserves for trial execution. However, it is possible that this absence of evidence was the result of the passive nature of task episodes in these experiments. Participants were merely biased to construe the set of trials as an episode and did not have to actively do something with the task episode. Hence, the difference in the magnitude of the program between 5 and 3 trial episodes may not have been large enough to cause discernible depletion of cognitive reserves available for component trial execution.

**Fig. 7:**
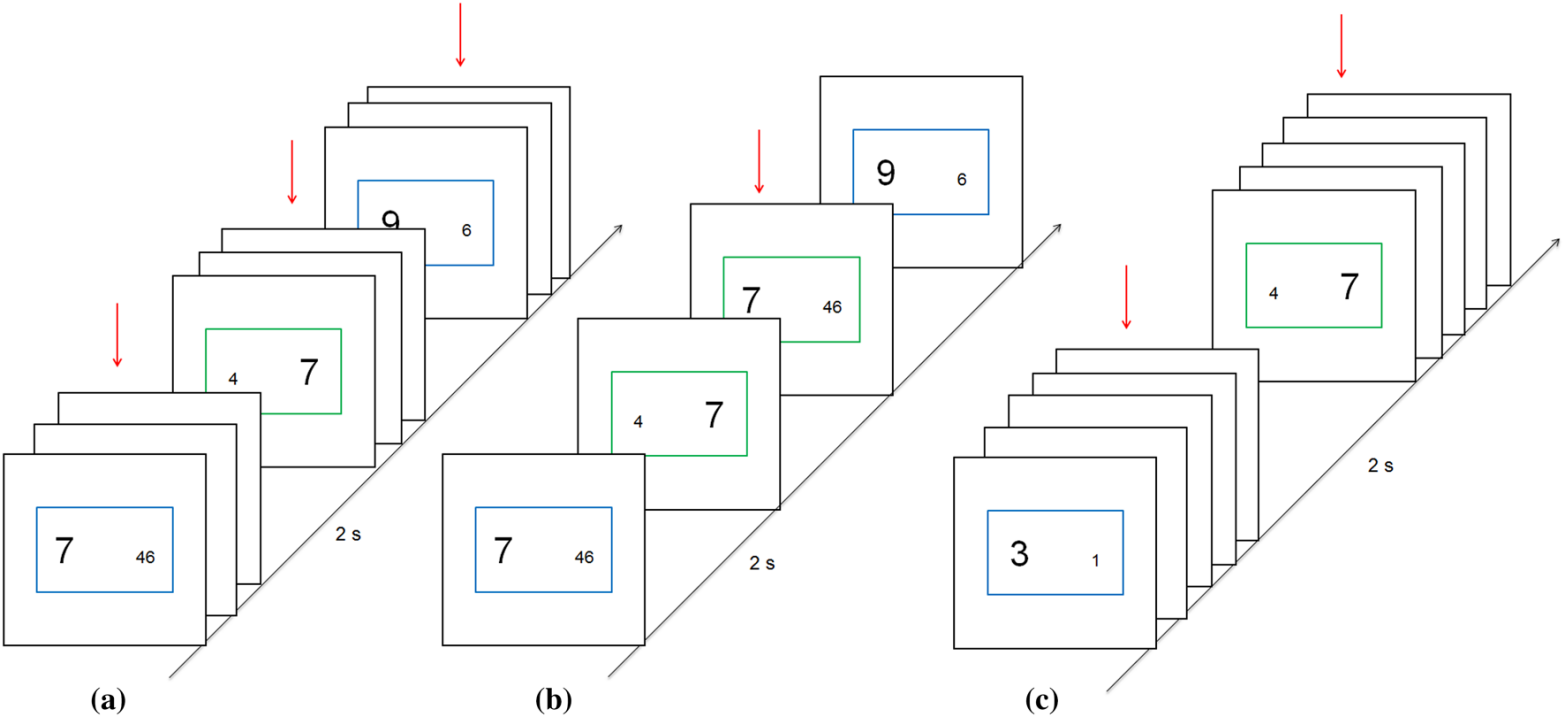
There were three kinds of blocks 3-trial-short, 5-trial-short and 3-trial-long. iTi within an episode in the first two were 0 s and in the third 2 s. Inter-episode interval was 2 s in all conditions. To ensure that the trials were actively executed as one episode participants had to keep a count of trials they had executed and then press an additional ‘task end’ key at the completion of each episode. (Color figure online)

In the current experiment task episodes had to be actively executed. Participants had to keep an active count of trials they had executed and press a ‘task end’ button at the end of each episode made of 3 or 5 trials. Trials were identical to those in experiments 1–3. They were executed in three different kinds of task episodes—3-trial-short, 5-trial-short and 3-trial-long (Fig. 7). In the first two there was no iTi within an episode and the next stimulus ensued immediately after response of the previous trial, whereas in the third (i.e., the 3-trial-long episodes) the iTi was 2 s. Inter-episode interval was 2 s in all task episode blocks. Hence, trial 1 of all three task episodes were preceded by the same iTi.

We made two predictions. First, if the program subsumes and organizes cognitive processing during the entire task episode then its magnitude may be related not just to the number of component trials but also to the temporal duration of the task episode. Trial 1 RT of 3-trial-long episodes may, therefore, be slower than those of 3-trial-short episodes in spite of both consisting of three trials. Second, maintaining programs related to longer episodes may require more cognitive reserves, causing a greater decrease in the amount of reserves available for individual trial execution during longer episodes. Consequently, not just trial 1 but subsequent RTs of 5-trial-short episodes will also be slower than the corresponding trials of 3-trial-short episodes. Note that such trials of 3-trial-long episodes could not be brought into this comparison, because these were preceded by a long iTi of 2 s, while those of 3 and 5-trial-short episodes followed immediately after the previous trial.

### Methods

Stimuli and trial rules were identical to Experiment 1. The key differences in this experiment concerned iTis and the additional requirement to press a ‘task end’ button (key ‘Z’ on QWERTY keyboard) at the completion of each task episode. In 3-trial-short and 5-trial-short episodes, the stimuli for the next trial followed immediately after the response to the previous trial (Fig. 7a, b). Within 3-step-long episodes the onset of the next trial occurred 2 s after the response to the previous trial during which the monitor was blank (Fig. 7c). In all conditions there was a 2 s delay between the end of an episode and the beginning of the next, hence trial 1 of all task episodes was preceded by identical iTis. The experiment consisted of blocks made of 60 trials. Nineteen Participants (11 females) completed 30–40 blocks. Each of these blocks consisted of one of the three task episodes (i.e., 20 task episodes in 3-trial-short and 3-trial-long blocks, and 12 task episodes in 5-trial-short blocks). The type of task episode to be executed in the block was cued by the instruction screen at the beginning of the block. Different task episode blocks were randomly interleaved with each other.

### Results

As predicted trial 1 RT of 3-trial-long episodes was slower than trial 1 RT of 3-trial-short episodes [paired *t*_18_ = 2.9, *p* ≤ 0.01, 95% CI of difference = (17, 103), Cohen’s *d* = 0.67]. Thus, the magnitude of the episode-related program was affected not just by the number of constituent trials or steps but also by the temporal duration of the episode. Second, difference in RTs between 3-trial-short and 5-trial-short episodes was not limited to trial 1, instead RTs on all trials of 3-trial-short episodes was slower than the corresponding trials of 5-trial-short episodes. Hence, the main effect of episode was significant (*F*_1,18_ = 10.5, *p* = 0.004) but its interaction with serial position was not (*F*_2,36_ = 0.4, *p* = 0.6), suggesting that maintaining larger program did decrease the amount of cognitive reserves available for trial execution. In other aspects results were identical to the previous experiments (Tables 11 and 12).

**Table 11:** Mean reaction times (ms) and accuracies (%) across the switch and repeat trials of the three task episodes

	3-trial short			3-trial long			5-trial				
	1	2	3	1	2	3	1	2	3	4	5
Sw	1090 ± 37 97 ± 0.6	947 ± 20 95 ± 1.2	979 ± 36 96 ± 1.1	1140 ± 45 97 ± 0.9	994 ± 30 96 ± 1.1	988 ± 30 96 ± 0.8	1144 ± 57 96 ± 1.3	1003 ± 26 97 ± 1.4	1012 ± 23 95 ± 1.2	1017 ± 30 96 ± 0.8	1039 ± 33 95 ± 1.1
Rp	1102 ± 34 94 ± 0.9	805 ± 30 97 ± 1.0	823 ± 25 97 ± 1.1	1140 ± 62 95 ± 1.1	928 ± 32 96 ± 0.8	939 ± 35 95 ± 1.1	1147 ± 61 94 ± 1.2	849 ± 37 97 ± 1.2	852 ± 31 98 ± 1.1	881 ± 30 98 ± 1.1	900 ± 21 97 ± 1.1

**Table 12:** Serial position: repeated measures ANOVA looking at the main effect of the position of trial within the episode on RT (and accuracy in parenthesis)

Effect	*df*s	*F*	MSE	*p*
Serial position (three short)	2,36	25 (1.3)	553,841 (1.4)	< 0.001 (0.3)
Rule switch (three short)	1,18	26 (0.2)	260,443 (0.2)	< 0.001 (0.6)
Rule switch × serial position (three short)	2,36	20 (7)	82,326 (0.005)	< 0.001 (0.002)
Serial position (three long)	2,36	40 (0.09)	397,508 (0.09)	< 0.001 (0.9)
Rule switch (three long)	1,18	2 (1.3)	41,402 (1.3)	0.18 (0.3)
Rule switch × serial position (three long)	2,36	2 (0.4)	10,948 (0.4)	0.13 (0.7)
Serial position (five short)	4,72	25 (2.4)	318,429 (0.002)	< 0.001 (0.06)
Rule switch (five short)	1,18	16 (4.5)	653,257 (0.006)	0.001 (0.04)
Rule switch × serial position (five short)	4,72	8 (3.4)	44,064 (0.003)	< 0.001 (0.01)

Rule switch: main effect of rule switch (switch vs repeat trials). Serial position × rule switch: interaction between the effects of rule switch and serial position

## General discussion

We showed that every time a series of otherwise independent and unpredictable trials was construed as a task episode (1) trial 1 had the longest RT, (2) which was longer before longer episodes, and (3) trial item-related switch cost was insignificant when the switch crossed construed episode boundaries. Results (1) and (2) showed that executing trial 1 required additional processing that was related not to the intrinsic demands of trial 1 but to the construed episode begun by this trial, and suggested that some episode-related cognitive entity had to be assembled every time a task episode was to be executed, and the magnitude of this entity was larger for larger episodes. Result (3) suggested that this episode-related entity had a hierarchical relation with cognitive configurations related to component trials, such that a change in this entity necessarily changed or ‘refreshed’ the trial-related configurations. As a result repeating and switching a trial type across episode boundaries became identical, i.e., in both cases trial-related configurations (e.g., trial-related set) had to be made afresh. We proposed that this entity was the program through which the task episode was executed as one entity.

The behavioral evidence of assembly of programs at episode beginnings provided by the current study is complemented by the neuroimaging evidence of their dismantling at episode completions. Completion of action sequences, perceptual episodes as well as task episodes typically elicit additional activity (Farooqui et al., 2012; Fox, Snyder, Barch, Gusnard, & Raichle, 2005; Fujii & Graybiel, 2003; Konishi, Donaldson, & Buckner, 2001; Zacks et al., 2001). This additional activity cannot be attributed to the intrinsic characteristics of the last event but only to the episode being completed (see Farooqui et al., 2012). Hence, for example, its magnitude and spread is affected by the hierarchical level of the episode completed (subtask < task).

If, as suggested by result (2) above, the program assembled at the beginning contains elements related to the entire duration of the ensuing episode, then as the episode is executed elements related to the completed parts of the episode may dismantle. Consequently, the cognitive load related to the program will be highest at the beginning when the program related to the entire episode is active, and will decrease as more and more parts of the episode get executed. fMRI of experiments 2 and 3 (Farooqui, Duncan, & Manly, 2018) showed that in neural regions known to deactivate in response to cognitive load (e.g., the Default Mode Network, Fox et al., 2005) beginning a task episode elicited a strong deactivation that then gradually decreased, with activity returning towards baseline, as sequential steps of the episode were executed.

One or more of the current observations have been made by previous studies in slightly different experimental contexts and interpreted differently. However, even those observations may be better explained by the thesis that all task episodes are subsumed and executed through programs. Execution of predictable sequences of trials across different studies has shown all of the three key results of the current study (e.g., Schneider & Logan, 2006, 2015). Though such studies frequently explain these in terms of the hierarchical nature of task-sequence representation in working memory. The presence of these very same observations in absence of such task-sequence representations suggests that these signs may be related to the execution of extended behavior as one task entity and not, per se, to the task-sequence representations.

It may be claimed in this regard that the knowledge that the episode consists of 3 or 5 trials in the current study was the task-sequence representation. This, however, does not explain why trial 1 RT remained high even when the same episode was iteratively executed. It is unlikely that this knowledge and its corresponding representation was forgotten at the end of every episode and had to be recalled afresh for the next iteration. This view can also not explain why recalling that the episode will be long or consist of five trials take longer than recalling that episode will be short/consist of three trials. Finally, this view can not explain why beginning a longer duration 3-trial episode take longer than beginning an identical 3-trial episode that is of shorter duration (experiment 6).

It can also be claimed that what are hierarchically stored in memory, and captured through constructs like tasks and goals, are not just linguistic representations, but include memories of past task and goal executions (e.g., Jacoby & Brooks, 1984; Logan, 1988; Medin & Schaffer, 1978; Neill, 1997). It is possible that instances of task execution generate a whole host of explicit and implicit/procedural memories (e.g., Jacoby & Brooks, 1984) organized around the goal (e.g., Logan, 1988), with re-execution strengthening the common elements across different iterations of the task episode executions and whittling away those that were specific to individual instances, thus creating memories of task execution. We agree with this account, and as we explain below, think that the execution of task episodes may occur by utilizing such past memories to assemble executive commands in a program for the current execution.

Another set of past studies have investigated what they call ‘restart cost’ using designs similar to that of experiments 2 and 3 (e.g., Gopher et al., 2000; Mayr, Kuhns, & Hubbard, 2014; Poljac, Koch, & Bekkering, 2009; Wylie & Allport, 2000). In these studies small runs of trials (with shorter iTis) are separated by longer iTis. The RT on the trial following longer iTi was delayed and was interpreted as a ‘restart’ cost. It was thought that the longer gap breaks the task item-related representations being maintained in WM, and the restart cost is related to the additional time taken to build these representations from long-term memory (Altmann & Gray, 2002), or to the interference from various task item-related long-term memory traces during this working memory updating (Mayr et al., 2014).

Two results from the current study suggest that this ‘restart cost’ may be related to the ensuing episode and not to the individual trial following the longer gap. First, this cost was related to the length of the task episode that ensued after the break (hence e.g., trial 1 RT for 5-trial episode > 3-trial episode), suggesting that what was assembled was not only related to the trial item being executed after the break, but to the larger task episode ensuing forth. Likewise, Poljac et al. (2009), noted that this cost was longer before more complex trial runs that had unpredictable trial rule switches compared to runs with predictable switches. Second, here in Experiment 1 there was no extended interval before the onset of each episode and yet ‘restart’ costs equivalent to those seen in Experiments 2 and 3 (where there was a gap) were observed. This suggested that it was the end of the conceived task episode, rather than the presence of a longer gap, that required a fresh assembly of cognitive entities. Plausibly, in the design of the ‘restart cost’ studies the period between two longer iTis became one task episode, and the ‘restart cost’ was related to the assembly of programs at the beginning of these episodes.

In summary, the current and related studies (Farooqui et al., 2012, 2018) suggest that purposive behavior is executed through programs related to the construed task and goal identities. These programs are assembled at the beginning of task episode execution, manifesting in additional delays at the beginning of episodes. They contain elements related to the entire duration of the episode, hence longer times are needed to begin longer episodes. They subsume the execution of the task episode causing the cost of switching component task items to be present only within an episode and to be undetectable when such switch occurs across episode boundaries. These programs dismantle gradually as parts of the episode are executed causing gradual change in related activity across the duration of the episode (Farooqui et al., 2018). Completion of the episode dismantles them completely and elicits widespread activity (Farooqui et al., 2012). Further insights into the content of such programs will have to await further studies, what follow are our current speculations.

### Relation to existing models of hierarchical behavior

Most attempts at modeling hierarchical behavior follow the assumption that execution of hierarchical behavior requires a parallel hierarchy of cognitive processing units (Cooper & Shallice, 2000; Estes, 1972; Norman & Shallice, 1986; Rumelhart & Norman, 1982). Typically, these models have discrete units for temporally smaller, lower level acts (e.g., ‘pick spoon up’) which are activated by units corresponding to temporally longer, higher level acts (e.g., ‘add sugar from packet’), which in turn are activated by the unit related to the entire task/goal (e.g., ‘prepare coffee’). Such models, however, can only work for situations, where identity and position of component steps of the task are explicitly known. In addition, the deterministic relation between their higher and lower level units creates a problem for generalizing actions across different task contexts as well as utilizing shared aspects of related actions. For example, across different coffee making situations sugar may be added before or after milk. Because the lower level acts making up the task of preparing coffee have fixed identities and ordinal positions, a small change such as this may require a totally different coffee making routine. In contrast to these models, the current study evidenced hierarchical cognition in situations, where higher level entities did not determine the identity and position of lower level steps.

Botvinick and Plaut (2004) suggested that a hierarchically organized processing system is not a prerequisite for hierarchical tasks. Instead, their model had perceptual input layer connected to the motor output layer through an internal hidden layer with recurrent connections between its component units. This allowed the units of this layer to not only link the perceptual units to motor units but to also represent the current behavioral context generated by the previous step. This got integrated with incoming information from the perceptual layer to choose the correct context appropriate next step. Through practice, the model learnt to represent the sequential structure of the task in the patterns of activations across units of the internal layer.

Contrary to the predictions of this model, the current results suggested that even behavioral sequences that have no external hierarchical structure or pre-existing internal hierarchical task representations get hierarchically executed if those sequences are conceived as defined task entities. Because this model executes one step at a time, in absence of any entity that corresponds to the overarching task episode, it cannot predict long step 1 RTs that are longer before longer task episodes. Likewise, because processings related to individual steps are not subsumed by anything related to goal or task episode, this model is unlikely to show absent step-related switch costs at episode boundaries.

### Programs

Existing accounts of cognition have proposed constructs like plans, scripts, schema and frames that subsume and control extended behavior but can be selected and instantiated as one entity (Cooper & Shallice, 2000; Miller et al., 1960; Minsky, 1974; Schank & Abelson, 1977). While the knowledge of the identity and sequence of component steps is a requisite in these accounts, the current study suggested that even unpredictable task episodes, where such knowledge is absent, are executed through subsuming cognitive entities that correspond to extended behavior. Such entities may be better thought of as embodying the executive commands for organizing, controlling and executing task episodes, and not as mere representations of the steps to be executed.

Hierarchical instantiation of executive commands is well known in motor cognition (Henry & Rogers, 1960; Keele, Cohen, & Ivry, 1990; Rosenbaum et al., 1983; Rosenbaum, Cohen, Jax, Weiss, & van der Wel, 2007). Motor actions typically consist of a sequence of smaller acts, e.g., articulating a word may consist of a sequence of phonemic articulations. Instead of individually instantiating higher level executive commands for each of these component acts, a motor program embodying the commands for the entire sequence is instantiated in one-go. This then unfolds across time into the seamless chain of small acts making up the overall action. While motor programs have typically been characterized in situations, where behavior seems to get executed ballistically, programs in general need not be limited to such instances.

When participants are given a memorized list to execute (e.g., CCSS, where C and S may, respectively, stand for color and shape decisions to be made across sequential trials), such that only the sequence of task items to be executed across time, and not the actual sequence of motor acts to be made, is known in advance, the behavior evidences a cognitive program, and not merely a recall of the sequence in working memory. Hence, not only is the item 1 RT high, it remains high even when the same sequence is iteratively executed, suggesting that something additional has to be done to the sequence already in working memory. Furthermore, this item 1 RT correlates with the number of item-level switches in the sequence (e.g., Schneider & Logan, 2006; Desrochers & Badre, 2015). Again, merely recalling a sequence like CSCS (with three item-level switches) need not take longer than a sequence like CCSS (with one item-level switch), but prospectively preparing for item switches to come may lead to longer item 1 RTs. The cognitive entity assembled at trial 1 thus embodies prospective control-related commands that will be needed during the ensuing episode, and has to be reassembled every time the episode has to be executed, even when the same episode is being iteratively executed.

Such programs related to memorized task-sequence execution did not directly translate into behavior, instead they translated into the sequence of rule-related cognitive set changes through which the correct motor act was selected in response to the stimuli (see discussion of Schneider & Logan, 2006). Analogously, the program in the current study could have translated into a sequence of control-related cognitive changes that facilitated the search for the correct rule-related cognitive set through which the correct motor act was subsequently selected.

The instantiation of attention and control is always linked to the goal in operation. Goals, typically, require some extended period of thought and behavior (i.e., task episodes) for their completion. It is possible that the executive commands for instantiating the myriad higher level goal-related control changes in cognition for a task episode is executed in one-go. If, for example, the goal requires searching for visual targets across 20 s, the higher level commands that instantiate the search and its related attentional changes in the brain need not be instantiated through separate commands every millisecond or every second. It is possible that these may be achieved through a program that subsumes the search period bringing about relevant changes across time.

Every task episode requires organization of cognition across time. Various irrelevant processes and representations need to be cleared out and maintained in abeyance across time so that they don’t compete for cognitive reserves and disrupt task execution. Various task-relevant learnings, memories, skills, dispositions, knowledge and expectancies, and the corresponding configurational changes in various perceptual, attentional, mnemonic, and motor processes may be brought to fore (Bartlett, 1932; Logan & Gordon, 2001; Mayr & Keele, 2000; Miller et al., 1960; Rogers & Monsell, 1995). The program may achieve these widespread changes across time. For example, in current task episodes processing related to mind-wandering, ongoing unconscious goals, task-irrelevant sensory and motor processing etc. had to be relegated. At the same time, the predictiveness of the episode was to be utilized to make anticipatory changes; e.g., knowledge that responses would be right handed, visual attention limited to area around fixation, along with an implicit idea of iTis, may have been used to increase preparations and attention at times when a stimulus was expected and decrease when iTi was expected.

### Programs and routines in cognition

Entities that are selected and instantiated as one unit but correspond to a sequence of processes and actions have been proposed and evidenced in other domains. Ullman (1984) suggested that the immediate and effortless perception of spatial properties and relations of objects may be achieved through automatic instantiation of a visual routine—a fixed set of elemental processes on early visual representations (for a related construct see Sprites, Cavanagh, Labianca, & Thornton, 2001). Logan (1999) suggested that attention during perceptual tasks be considered as a routine (attentional routine) consisting of the set of processes that intervene between perception and reaching a goal relevant propositional conclusion, e.g., during identification of object X this routine would intervene between early perceptual processes and the reaching of conscious conclusion that the ‘visible object is X’. Component motor acts of a larger motor action come about through a common program assembled at the beginning of execution (Henry & Rogers, 1960; Schmidt, 1975). Fan et al. (2012) found that creating a means-ends relation between two sequential tasks created an additional delay in the execution of the first task, which they interpreted as related to the time taken by the supervisory control system in chaining subunits into longer goal-directed behavior (see also Sacker & Dehaene, 2009). Botvinick et al. (2009) proposed that extended sequence of behavior (e.g., ‘open laptop’, ‘mouse to browser icon’, ‘double-click’, ‘enter URL’, among others) may be chunked into larger routines (‘check email’) through hierarchical reinforcement learning, wherein the reward signals strengthen the entire sequence of behavior, and not just one act, allowing instantiation of the entire sequence as a routine. In comparison, current results suggested that abstract, unpredictable, higher level sequential behavior can be executed as one-routine in absence means-ends relation and without receiving hierarchical reinforcing reward signals if that behavior is construed as one task entity.

We suggest that our phenomenal experience of executing task identities (e.g., ‘prepare breakfast’, ‘boil water’) as a whole, and not of individually executing its very many components, may be real (Vallacher & Wegner, 1989). These typically correspond to an episode and consist of numerous sequential acts (‘prepare breakfast’, ‘boil water’). We suggest that our phenomenal experience of executing such task identities as a whole, and not of individually executing its very many components, may be real. At the beginning of task episodes we prepare for the entire episode as one unit. This preparation is embodied in a program. The actual contents of this program, and the prospective preparation it embodies, may depend on the familiarity and predictability of the task episode related to that goal. Completely predictable tasks may be prepared down to the level of individual motor acts to be made across time (e.g., Henry & Rogers, 1960; Rosenbaum et al., 1983). During less predictable task episodes like memorized task-sequence execution the program may involve preparing for the sequence of stimulus—response rules to be applied across time (e.g., Schneider & Logan, 2006). During unpredictable task episodes, the program may only instantiate goal-directed attention and other control processes, and their related changes in cognition that enable the search for implementation strategies that will culminate in goal completion.

## Conclusions

We showed that signs of hierarchical cognition, identical to that seen previously during the execution of memorized and predictable behavioral sequence, can be seen whenever any extended arbitrary segment of behavior is construed as one task episode, even when the components of that extended behavior are unknown and unpredictable. We suggest that task episodes, irrespective of the predictability of their component steps, are executed through programs that are assembled at the beginning and subsume and control the execution of the episode. We argue that these programs are the means of instantiating control across time and organizing cognition into goal-directed episodes.
